# Towards Precision Prognostication and Personalized Therapeutics through Proteomics

**DOI:** 10.3390/ijms24076361

**Published:** 2023-03-28

**Authors:** Enrique Santamaría

**Affiliations:** Clinical Neuroproteomics Unit, Navarrabiomed, Hospitalario Universitario de Navarra (HUN), Navarra Institute for Health Research, Universidad Pública de Navarra (UPNA), IdiSNA, Irunlarrea 3, 31008 Pamplona, Spain; esantamma@navarra.es

Next-generation proteomics has allowed the implementation of biomedical proteome research to uncover disease-affected protein expression profiles. It has also enabled the determination of protein localization, protein interactomes, posttranslational modifications and protein dysfunction in human diseases. Many pillars in personalized medicine, such as diagnostic improvements, drug screening, systems biology or bioinformatics, require the generation of quantitatively consistent proteomics data from translational animal models to human biospecimens to fill the information gap, making omics analysis actionable from a clinical perspective [1,2,3]. This Special Issue received multiple submissions, of which five original articles were accepted for publication. These contributions cover different phases of precision medicine in the context of proteomics: (i) discovery and quantitation of potential biomarker candidates (three articles), (ii) the proteostatic modulation and mechanisms of action of pharmacological compounds (one article) and (iii) the characterization of posttranslational modifications (one article).

Proteomics has emerged as a powerful approach with which to characterize the molecular composition of different biofluids, with the aim of discovering potential biomarkers. Yohannes E. et al. performed a label-free LC-MS/MS workflow to detect changes in the maternal blood proteome across pregnancy, using plasma across the three trimesters. Beyond the identification of proteins relevant for placentation, they observed protein signatures that highly correlate with gestational age. Orthogonal validation in an independent cohort revealed that plasma levels of Disintegrin and metalloproteinase domain-containing protein 12 (ADAM12), independently or in combination with Pregnancy-specific beta-1-glycoprotein 1 (PSG1) and Chorionic somatomammotropin hormone 1/2 (CSH1/2), can determine gestational age at any trimester with a period of +/−8 days, in contrast with the +/−14 days obtained using an ultrasound technique [4]. In oncology, the sputum is an enriched protein source that is highly useful in achieving early diagnosis of lung cancer [5]. In this context, Arenas-De Larriva MDS et al. optimized a diaPASEF mass-spectrometry acquisition mode to detect and identify lung cancer protein biomarkers to discriminate different cancer subtypes and controls. From a functional point of view, differential sputum proteome maps were clearly linked to activation of inflammation, detecting acute-phase and complement cascade protein intermediates. Interestingly, although the proposed biomarker panel is in need of further validation, feature selection through a sparse partial least squares discriminant analysis (sPLS-DA) revealed a coherent separation between cases and controls with high sensitivity and specificity [6]. This type of workflow implemented with low-invasiveness samples may be an ideal option to be applied in screening and diagnostic programs with the aim of identifying new pharmacological targets and potential biomarkers, as well as improving the tumor clinical management. In the field of cardiovascular biomedicine, the deployment of proteomic approaches is mainly focused on the discovery of circulating protein biomarkers of heart diseases and the elucidation of disease-associated mechanisms and potential therapeutic targets in cardiovascular tissues [7,8]. Nav1.5 protein corresponds to the sodium channel protein type 5 subunit alpha (SCN5A) of the human cardiac voltage-gated sodium channel. This protein mediates the voltage-dependent sodium ion permeability of excitable membranes in cardiac muscle, participating in the excitation–contraction coupling cascade in cardiac cells. Its alteration has been observed in multiple cardiovascular pathologies, and only relative quantities have been reported using different techniques such as Western-blot, immunofluorescence and patch-clamp. Moreover, the Nav1.5 protein measurement presents technical challenges associated with its high molecular weight (220 KDa), presence of 24 transmembrane domains and multiple posttranslational modifications. Adams SL et al. established a method for performing an absolute quantitation of Nav1.5 copy numbers in a biological matrix [9]. This method is based on targeted mass-spectrometry quantitation (parallel reaction monitoring—PRM) using four peptide sequences derived from Nav1.5 in CHO cells. Due to this workflow’s capacity to be simultaneously multiplexed for measure multiple proteins, authors propose the absolute quantitation of different ion channels and associated proteins by PRM as a further step towards an implementation of computer modeling to push both predictive and preventative cardiac health at a population scale. Martínez-Martínez E et al. applied a quantitative proteomic approach to explore the obesity induced in a rat model at the cardiac level and the beneficial effects induced by a mitochondrial antioxidant (MitoQ). In the absence of functional cardiac alterations, transthyretin (TTR) was increased at cardiac and plasma levels in obese animals. Moreover, TTR induced profibrotic events and ER stress activation through mitochondrial oxidative stress in cardiac cells, suggesting a possible novel approach to TTR-related diseases [10].

Post-translational modifications (PTMs) are critical molecular mechanisms that dynamically regulate protein functions in a temporal and spatial manner. The localization of PTMs on specific drivers of neurodegeneration is a valuable step towards the elucidation of biochemical and structural derangements that accompany the progression of neurological disorders [11]. Through a palmitoyl-proteomic approach, Cervilla-Martínez JF et al. detected a differential palmitome in the cortex from Parkinson’s disease (PD) subjects, reinforcing the alteration of lipid metabolism in this disease. Part of this altered palmitoylated proteome pointed not only to protein interactors of relevant PD-related proteins. but also to fibrinogen and cytoskeletal proteins as new targets linked to the neurodegenerative process that accompany the progression of PD [12].

Although no papers proposed for this Special Issue primarily addressed the proteomic consideration of sex-/gender-specific pathophysiology as an essential part of precision prognostication and personalized therapeutics [13], these original works demonstrate the capacity of different proteomic methodologies to generate novel pieces of biomedical knowledge at fluid, cellular and tisular level, which are needed to fill the gaps in the definitive consideration and implementation of precision proteomics in the era of personalized medicine. 
